# The Role of Muscle Biomarkers in Adolescent Idiopathic Scoliosis

**DOI:** 10.3390/jcm12247616

**Published:** 2023-12-11

**Authors:** Federico Roggio, Bruno Trovato, Martina Sortino, Maria Pia Onesta, Luca Petrigna, Giuseppe Musumeci

**Affiliations:** 1 Department of Biomedical and Biotechnological Sciences, Section of Anatomy, Histology and Movement Science, School of Medicine, University of Catania, Via S. Sofia n 97, 95123 Catania, Italy; federico.roggio@unict.it (F.R.); bruno.trovato@phd.unict.it (B.T.); martina.sortino@phd.unict.it (M.S.); luca.petrigna@unict.it (L.P.); 2 Sport and Exercise Sciences Research Unit, Department of Psychology, Educational Science and Human Movement, University of Palermo, Via Giovanni Pascoli 6, 90144 Palermo, Italy; 3 Spinal Cord Unit, Cannizzaro Hospital, 95100 Catania, Italy; mariagiuseppa.onesta@aoec.it; 4 Research Center on Motor Activities (CRAM), University of Catania, Via S. Sofia n 97, 95123 Catania, Italy; 5 Department of Biology, Sbarro Institute for Cancer Research and Molecular Medicine, College of Science and Technology, Temple University, Philadelphia, PA 19122, USA

**Keywords:** adolescent idiopathic scoliosis, biomarkers, GWAS, protein, single-nucleotide polymorphism

## Abstract

Adolescent idiopathic scoliosis (AIS) is the predominant orthopedic disorder in children, affecting 1–3% of the global population. Research in this field has tried to delineate the genetic factors behind scoliosis and its association with heredity since AIS is considered a polygenic disease and has different genetic and epigenetic factors. The current study conducted a narrative review of the literature, focusing on biomarkers in the pathophysiology of muscle in AIS patients. Articles were collected from Scopus, Pubmed, and Web of Science. The key screening parameters were scoliosis classification, sampling, and the biomarkers evaluated. This review emphasizes potential key mechanisms and molecular regulators in muscle tissue. While there has been limited focus on the proteins contributing to muscle changes in AIS, significant attention has been given to genomic studies of single-nucleotide polymorphisms, particularly in LBX1. Despite these efforts, the exact causes of AIS remain elusive, with several theories suggesting genetic and hormonal factors. This review identified critical protein biomarkers such as Gi-protein alpha subunits, fibrillin-1 and -2, and various differentially expressed proteins, which may be linked to muscle alterations in AIS. This field of research is still limited due to a lack of homogeneity in the distinction of patients by groups and curve severity. Although the pathophysiology of AIS is still unclear, molecular research is important to guide the treatment of AIS before achieving skeletal maturity, thus avoiding serious problems associated with posture changes and low quality of life. In the future, a more comprehensive synergy between orthopedic and molecular research might ameliorate the diagnosis and treatment of AIS patients.

## 1. Introduction

**Figure 1:**
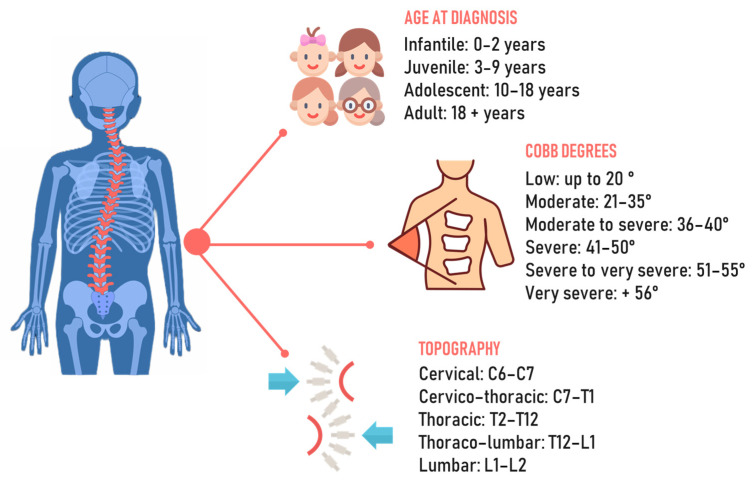
Chronological, angular, and topographic classification of scoliosis.

Spinal deformities, particularly scoliosis, represent the most common orthopedic deformities in the pediatric population, with idiopathic scoliosis being the most prevalent form [1]. The spine is characterized by structural changes in the vertebrae that result in rotation and translation relative to the normal body axis [2]. Scoliosis is defined as idiopathic because, until now, no identifiable cause of it has clearly been recognized. Theories include a broad idea of genetic and hormonal factors or more specific causes related to the asymmetric growth of the musculoskeletal system [3]. According to the age of diagnosis, it can be classified as infantile when it occurs between the ages of 0 and 3 years, juvenile when occurring in the age range of 4–10, adolescent at ages 10–18, and adult when diagnosed after the age of 18 [4]. It is important to properly consider the age of onset, since different age ranges have unique physiological aspects to consider, such as bone, muscle, and hormonal maturation (Figure 1). The diagnostic approach comprises clinical analysis using the forward bending test and a scoliometer, followed by X-rays to identify the degree of curvature of the scoliosis. In addition to diagnosis, these methods allow us to observe the condition’s progression and choose the most suitable approach for treatment. According to the Scoliosis Research Society, scoliosis is diagnosed as such only when there is an axial rotation of the vertebrae and the curve is greater than the Cobb angle [5].

Scoliosis screening programs are important because early detection of scoliosis can slow its progression and prevent additional musculoskeletal disorders [6]. As observed in a previous investigation, the presence of clinical signs of scoliosis among Italian high school adolescents was 36% in the sample examined [7], highlighting the lack of adequate screening programs.

Scoliosis diagnosis involves the forward bending or Adam’s test, scoliometer use for trunk rotation measurement, and radiography, the gold standard for identifying and quantifying spinal alterations [8,9,10]. To avoid exposure to repeated X-rays in a short period of time, different non-invasive methods have been proposed to help clinicians to select the most appropriate treatment to prevent scoliosis progression, such as Moiré topography [11], infrared thermography [12], rasterstereography [13], 3D scanner [14], and 3D ultrasound imaging [15]. These methods cannot diagnose AIS, but their ease of use can highlight possible improvements or worsening at follow-up, helping to better tailor treatment. Monitoring AIS is necessary as progression may be aggressive, resulting in the need for surgery if scoliosis is not adequately treated.

These different methods are a valid source of support; however, research has been trying to classify the genetic factors behind scoliosis [16] and its association with heredity, first described in 1934 by Garland [17]. He observed that the frequency of scoliosis in first-degree relatives is much higher than in the general population. Epidemiological and genetic studies consider AIS a polygenic disease, describing an increased risk of onset of scoliosis due to different genetic and epigenetic factors [18,19]. Different loci have been identified as strongly associated with susceptibility to AIS, but this clinical approach is still limited to supporting the prediction of scoliosis or its progression [20], rather than diagnosis before its onset. Since genetic markers represent valid prognostic tools, it is important to promote studies on the classification of scoliosis and its progression to help clinicians manage personalized treatments.

The exploration of molecular biomarkers in AIS has yielded significant insights into its pathogenesis and opened potential opportunities for early diagnosis and treatment. For instance, a study by Seki et al. [21] revealed an association of ligamentum flavum hypertrophy with AIS progression, identifying the ERC2 and MAFB genes as significant contributors to this phenomenon. These genes were linked to increased expression of collagen by ligamentum flavum cells, highlighting a molecular pathway that could be targeted for therapeutic intervention. Another pivotal study, conducted by Sun et al. [22], explored potential metabonomic biomarkers for AIS, identifying seven differential metabolites, including PC(20:4), 2-hexenoylcarnitine, and beta-D-glucopyranuronic acid. These metabolites indicated a disrupted lipid metabolism in AIS, suggesting that lipid metabolism plays a significant role in the pathogenesis of AIS. This discovery sheds light on the development of diagnostic biomarkers and also provides a deeper understanding of metabolic alterations in AIS.

The aim of this study is to review the current literature on the biomarkers involved in the pathophysiology of muscle in patients with AIS, highlighting some potential key mechanisms and intrinsic molecular regulators within muscle tissue.

## 2. Materials and Methods

This narrative review considered all articles analyzing AIS through biomarkers involved in the musculoskeletal system. Studies were included if they met the following inclusion criteria: case–control design, diagnosis of AIS, description of genetic and epigenetic factors associated with AIS muscle disorders. Articles were retrieved from Pubmed, Scopus, and Web of Science and were downloaded on EndNote 20 (EndNote 20 desktop version, Clarivate, Philadelphia, PA, USA) [23], and screened in order to identify significant information. Relevant data extracted from selected studies included: number of patients, ethnicity, data collection time window, age, scoliosis classification, method of collection of analyzed samples, biomarker considered, results, and conclusions. We checked if authors correctly addressed the clinical examination of participants before biomarker analysis in order to ensure the correctness of the results. We discussed the articles narratively.

## 3. Results

**Figure 2:**
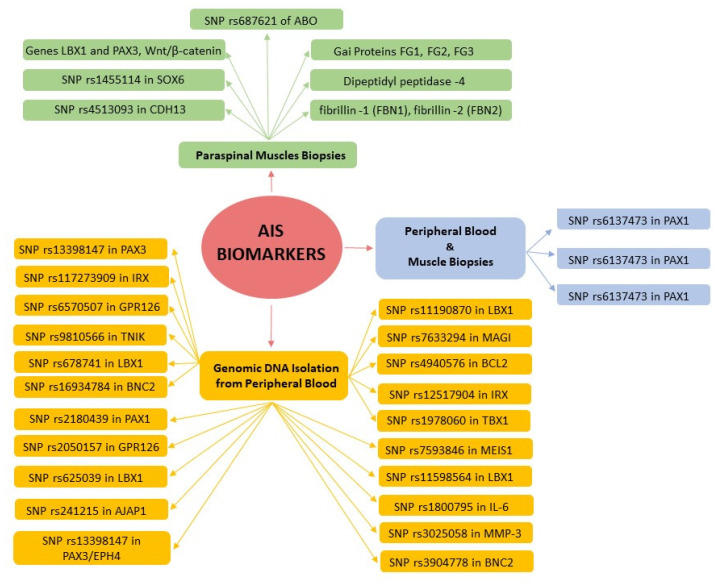
Mind map of the three different areas of biomarker research.

All the articles examined discussed AIS, specifically ‘adolescent’ AIS. Researchers based their results on the collection of biological samples such as peripheral blood, peripheral mononuclear cells, and muscle biopsy. Of these articles, four investigated alterations in specific proteins, while nine articles analyzed the presence of single-nucleotide polymorphisms (SNPs) related to specific genes (Figure 2). All articles correctly reported the initial orthopedic evaluation of patients with appropriate diagnostic tools, i.e., clinical tests and X-rays, in order to avoid incorrect data collection due to the absence of AIS. All the articles except two reported the severity of scoliosis based on the Cobb angle classification. The age range was 9–18 years, which ensured that the studies included precisely classified the adolescent age group [4]. The characteristics of the studies are summarized in Table 1.

### 3.1. Protein Studies

We found four studies that analyzed proteins from myocytes, peripheral blood, and paraspinal muscle biopsies; however, they all analyzed different proteins. The results concerning proteins showed that the Gαi protein fG1 endophenotype was equally present in mild and severe AIS (*p* = 0.545), FG2 was overrepresented in the AIS group with Cobb angle > 40° (*p* = 0.0005), and FG3 was overrepresented in the AIS group with Cobb angle < 40° (*p* = 0.0001) [24]. A rare variant of the proteins fibrillin-1 and fibrillin-2 was present in the paraspinal muscle of European (*p* = 0.0012) and Chinese (*p* = 0.0376) AIS cohorts [25]. The expression of Dipeptidyl peptidase-4 was downregulated in AIS patients (*p* = 0.032) [26] and finally, muscle-related proteins AIM1L, SOX2, WDR7, and DNM3 were downregulated in the AIS population while ACTA1, TMP2, ILK, PKM, TLN1, CTTN, CALM1, TPM1, TPM3, TPM4, CALR, VCL, MYLK, MYL6, MYL12A, FLNA, WDR1, and ENO3 were upregulated.

### 3.2. Single-Nucleotide Polymorphism Studies

The background of the articles that studied the genetic variation of specific genes is based on genome-wide association studies (GWAS) [26]. Some of these articles focused on the same genes but did not always choose the same single-nucleotide polymorphism (SNP). The LBX1 gene was the most investigated in the considered articles. The odds ratio (OR) for the SNP rs11190870 of LBX1 was 1.70 (1.42–2.04) [27] and 1.56 (1.21–2.01) [29]; for the SNP rs678741, it was 1.67 (1.29–2.15) [27] and 1.42 (1.26–1.60) [34]. Only Gao et al. [27] evaluated two more LBX1 SNPs, rs625039, with OR = 1.49 (1.23–1.80), and rs11598564, with OR = 1.52 (1.27–1.83). Xu et al. [35] evaluated mRNA and found a lower expression of LBX1 (*p* = 0.03) in the muscles of the concave side than the convex side. They also evaluated the mRNA of the PAX3 gene, finding a lower expression (*p* = 0.001). The only investigated SNP of PAX3 was rs13398147, on which conflicting results were reported, because Man et al. [29] found an OR = 0.88 (0.65–1.18) while Xu et al. [34] found an OR = 1.46 (1.27–1.49), both investigating this gene in Han Chinese females. For the genes AJAP1 and BNC2, the results were in conflict. Considering the SNP rs241215 of AJAP1, Man et al. found an OR = 1.11 (0.83–1.47) while Xu et al. provided an OR = 0.75 (0.66–0.86). For the BNC2 gene, Man et al. analyzed the SNP rs3904778, finding an OR = 0.74 (0.57–0.96), while Xu et al. found an OR = 1.35 (1.18–1.55) for the SNP rs16934784. Two studies by Xu et al. evaluated three different PAX1 SNPs, reporting an OR = 1.17 (1.09–1.26) for rs6137473 [33], OR = 1.22 (1.14–1.30) for rs169311 [33], and OR = 1.25 (1.11–1.42) for rs2180439 [34]. The remaining SNPs were not comparable, as each article assessed different ones. Man et al. also evaluated rs12946942 in SOX9/KCNJ2 with OR = 0.82 (0.66–1.18) and rs6570507 in GPR126 with OR = 1.39 (1.07–1.79). Xu et al. found an OR = 1.24 (1.10–1.41) for rs2050157 in GPR126, OR = 1.39 (1.23–1.57) for rs4940576 in BCL2, OR = 1.23 (1.09–1.39) for rs7593846 in MEIS1, OR = 1.27 (1.12–1.44) for rs7633294 in MAGI1, and OR = 1.30 (1.15–1.47) for rs9810566 in TNIK. Liu et al. found no significant differences in the SNPs rs3025058 in MMP-3 and rs1800795 in IL-6 between AIS and the control group. Wu et al. [31] found a significant difference between AIS and control only in the SNPs rs687621 in ABO, OR = 0.87, and rs4513093 in CDH13, OR = 1.20 (1.04–1.40); no significant differences were found for rs1455114 in SOX6. Li et al. [36] analyzed only the SNP rs1978060 in TBX1, whose expression was lower in AIS patients than in patients with congenital scoliosis, OR = 1.12 (1.02–1.22); finally, Xia et al. [32] analyzed the SNPs rs12517904 and rs117273909 in IRX, with OR = 1.14 (1.02–1.26) and OR = 1.01 (0.40–2.56), respectively.

## 4. Discussion

**Figure 3:**
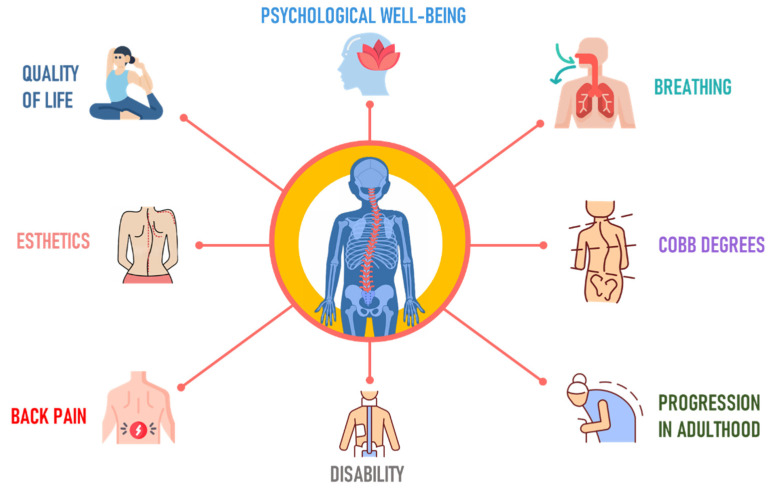
Goals in the treatment of adolescent idiopathic scoliosis.

Research on AIS is important in orthopedics due to its impact during musculoskeletal development. This condition can cause not just physical deformities [37] but also muscle imbalances [38] and diminished lung capacity, stemming from changes to the spine and ribcage [39,40] (Figure 3).

The term ‘idiopathic’ refers to the inability to identify a specific cause and is why research has been trying to map all possible genetic or epigenetic factors associated with AIS in recent decades. Other studies summarized the main characteristics of the relationship between scoliosis and some specific biomarkers. Zhu et al. [41] found that vitamin D deficiency may be involved in AIS pathogenesis because it affects the regulation of calcium phosphate metabolism in bones. Recent studies explore how leptin and ghrelin signaling might contribute to the changes observed in body mass index in AIS patients [42]. Leptin is mainly produced in white adipose tissue, while ghrelin is a polypeptide hormone regulating growth hormone secretion and bone formation. Recent research indicates a correlation between ghrelin and the abnormal cartilage development seen in AIS patients [43]. Wang et al. [44] conducted a meta-analysis on this topic and observed lower serum levels of leptin and higher serum levels of sOB-R and ghrelin in patients with AIS compared to a control group. Two systematic reviews analyzed the SNP rs11190870 of the LBX1 gene. Akbik et al. [45] found that this SNP is associated with AIS, although they also evaluated adults, while Chen et al. [46] conducted a meta-analysis of articles related to this SNP and found an OR = 1.69 and OR = 2.62 for the genotypes TC and TT, respectively. To the best of our knowledge, no other studies investigated muscle biomarkers specifically focusing on adolescents.

For the present narrative review, we focused on articles that correctly corresponded to our selected age range of AIS and that analyzed the biomarkers involved in muscle alterations. These articles aimed to achieve two primary objectives, highlighting different expression in patients with AIS and, moreover, considering a possible association between significant biomarkers and the severity of progression. Within the scenario of AIS marker research, two primary approaches exist: one focuses on variations in protein concentration in targeted areas such as spinal muscles, while the other relies on genomic analysis derived from blood samples. Regardless of these different methods, we posit that the diversity in the results could partially stem from the structural variability of scoliosis, which is reflected similarly in its molecular characteristics [47,48,49].

The alpha subunits of the Gi protein mainly inhibit cAMP-dependent pathways by inhibiting the activity of adenylyl cyclase and reducing the production of cAMP from ATP [50]. Akoume et al. [24] analyzed the contribution of Gi-protein-coupled receptor signaling in the pathogenesis of AIS. They classified patients with AIS into three endophenotypes (FG1, FG2, and FG3) and observed that the FG2 group was the most likely to develop severe scoliosis (Cobb angle > 45°) in both osteoblasts and myoblasts. Gαi_2_ is related to skeletal muscle hypertrophy and myoblast differentiation [51,52], so the differences found by Akoume et al. [24] in the three groups of Gαi proteins may be related to muscular imbalance due to postural asymmetry in scoliosis. Buchan et al. [25] postulated the same concept of muscle imbalance in their investigation of fibrillin-1 and fibrillin-2, a topic first broached by Miller et al. in 1997 [53]. While the FBN1 gene mutation is tied to Marfan syndrome [54], its association with skeletal muscle microfibrils suggests that rare variants in AIS patients could lead to vertebral and muscular structural changes due to altered elasticity. FBN2, which is known to regulate TGF-β (transforming growth factor-β) signaling [55], plays a role in the inflammatory response of skeletal muscle. It inhibits muscle regeneration and governs the remodeling of the extracellular matrix [56]. Probably, muscle alterations related to FBN1 and FBN2 and their activity on TGF-β may explain muscle and bone impairments in patients with AIS. Also, a study by Dai et al. [26] discussed muscle changes in AIS. They analyzed the concentration of dipeptidyl peptidase-4, a transmembrane protein that modulates insulin metabolism. The authors found that AIS muscles demonstrated reduced sensitivity to glucose and insulin, which could be linked to cell viability during myogenesis.

Regarding the analysis of SNPs, the studies we selected are mainly based on the GWAS protocol. LBX1 is an important regulator of muscle precursor migration [57] and was the most investigated gene in the present study. Two SNPs, rs11190870 and rs678741 [27], were found to have a higher OR for individuals with AIS compared to the control group, while the results of Man et al. [29] and Xu et al. [34], although significant, had a lower OR. LBX1 mRNA expression was also found to be lower in the muscles of the concave side compared to the convex one [35]. This gene was also the most investigated among the selected studies. Fan et al. [58] found an OR = 1.85 for the SNP rs11190870, which was higher than those reported in this systematic review. Similarly, Jiang et al. [59], Liu et al. [28], and Nikolova et al. [60] analyzed the same SNP providing an OR = 1.51, OR = 0.61, and RR = 1.37, respectively.

PAX3 is another gene providing conflicting results for the SNP rs13398147. Man et al. [29] found a lower OR of 0.88 than Xu et al. [34], who found an OR of 1.46, while in another study, the authors provided an OR = 1.48 [19], similar to that of Xu et al. [34]. Man et al. [29] and Xu et al. [34] were also in conflict regarding the SNP rs241215 (OR = 1.11 vs. OR = 0.75) of the gene AJAP1. Both authors also evaluated the genes SOX9/KCNJ2, GPR126, BCL2, BNC2, MEIS1, MAGI1, and TNIK, but they identified different SNPs, making them non-comparable. Wu et al. [31] found significant differences in two SNPs in the genes ABO and CDH13, while Li et al. [36] found a lower expression of the SNP rs1978060 in TBX1 in patients with AIS compared to patients with congenital scoliosis. Xia et al. [32] analyzed two SNPs in IRX, with a higher OR for rs12517904 but not for rs117273909. Liu et al. [61] evaluated an SNP of the IL-6 gene, reporting no significant association between this SNP and AIS. These results are in contrast to those of Nikolova et al. [62], who found the IL-6 gene as a modifier factor for idiopathic scoliosis. The study of Xu et al. [35] investigated the Wnt/β-catenin pathway. They found it had a lower expression on the concave side of scoliosis. These results are corroborated by Zhu et al. [63], who found an asymmetric expression of the Wnt/β-catenin pathway in the paraspinal muscles of patients with AIS.

We believe that the main characteristic to be addressed when conducting these studies is to correctly divide the sample based on scoliosis severity. This is an important aspect to consider because the diversity of results may still be related to the neglect of degrees of scoliosis. As highlighted, it is currently not possible to identify a specific cause of the development of AIS, as it still concerns a multifactorial condition. Although the authors we cited have greatly improved the progress in this field, the causes of AIS continue to elude research. While these steps elucidate the underlying mechanisms of AIS, we are hesitant to support the belief of Azeddine et al. [64] that these molecular studies could serve as a new AIS classification, replacing the current orthopedic classification. They perceive scoliosis through the lens of quality-of-life outcomes, thereby reflecting a more patient-centered approach. We know that the effectiveness of rehabilitative treatment is dependent on the stage of skeletal maturation. An earlier diagnosis leads to better treatment outcomes and potentially reduces the need for surgery [65]. We posit that early detection of warning signs via molecular studies can accelerate rehabilitative treatments, subsequently reducing the occurrence of postural alterations and improving quality of life.

Molecular differences could inspire new treatment strategies. Therapies targeting the Gαi protein, for instance, could potentially be developed to address the muscle imbalances caused by scoliosis’s postural asymmetry, thereby reducing the severity of the condition. Understanding the role of genes such as FBN1 and FBN2 in skeletal muscle microfibrils could pave the way for innovative treatment approaches. Targeted gene therapy could also potentially be used to correct these mutations and prevent the muscular and vertebral alterations associated with the disease. Furthermore, identifying specific SNP variations could guide genetic counseling and foster a personalized medicine approach, optimizing interventions based on a patient’s genetic risk. A blood test for these biomarkers could facilitate earlier detection and treatment of AIS, potentially mitigating disease progression and improving patient outcomes.

These results show a complex picture of genetic variability in individuals with AIS, with conflicting results and multiple genes and SNPs that may be associated with the disease. More research is needed to clarify these associations and their potential implications for the diagnosis, treatment, and management of AIS.

### Perspectives

Adolescent idiopathic scoliosis has been studied for many years; the first scientific findings date back to the mid-1800s [66], and research has always wondered about its etiology. The term idiopathic refers to the inability to find a clear etiology. Although there are very good non-invasive methods for diagnosing and predicting its progression, knowing this disease from a molecular point of view serves to clearly define its course and thus effectively treat it. Currently, many single-nucleotide polymorphisms, specific genes, and proteins have been found to be altered in scoliosis patients. Unfortunately, studies carried out on large samples have required long periods of data collection, leading to less than perfectly reliable results. The implementation of more advanced sequencing techniques like next generation sequencing, which allows us to sequence a whole genome in a short period of time [67], might help with the identification of new specific genetic variants. This approach could be beneficial for patients, allowing for a more specific tailoring of their treatment based on their genome, and provide insight on disease progression. A major limitation at present is the diversity of scoliosis in the world. Leaving aside the age of onset, a point we widely discussed, other specific elements such as the curve’s amplitude, its anatomical position, and whether there is one or two primary curves, could potentially impede the progression of molecular research into diagnostic biomarkers. Being able to classify scoliosis in a specific way could be a first step in accumulating more accurately identified biomarkers.

What this narrative review makes evident is the lack of a clear differentiation between groups and curves. Distinguishing Cobb angles is fundamental, as the musculoskeletal alterations resulting from a 15–20° Cobb angle scoliosis can never be the same as those from a 50° Cobb angle scoliosis. Consequently, the biological signals of the biomarkers involved in curve progression also vary. Similarly, anatomical location is an important factor because if during embryonic development each sclerotome starts to separate into a cranially located cluster and a caudally located cluster [68], genetic and epigenetic differences could be zone-specific. Therefore, the best way to improve research in this field is to follow a proper division of cases into the key factors discussed. This division can be constructive because it will allow all findings to be classified and then studied according to the specific aspects of scoliosis. Undoubtedly, both the molecular and orthopedic fields of scoliosis study need significant advancement. Current discoveries are enabling the quantification of the aggressiveness of the disease and the degeneration of the curve. However, having a precise categorization of patients could facilitate the selection of the most suitable treatment. This includes brace prescription, physiotherapy, choice of sport, and the necessity of surgery. Thus, the most effective result for the patient can be achieved.

We believe that the future of these studies lies in machine learning and big data analysis techniques [69,70]. The task of research at present must be to gather as much information as possible about this pathology, trying to correctly classify each of the mentioned aspects. Once this has been done, these data will have to be analyzed using specific computational methods capable of identifying connections that are not significant for humans but valid for machine learning models. If this happens, it will only take a few years and it will be possible to genetically describe not only scoliosis but thousands of other multifactorial diseases.

## 5. Conclusions

Adolescent idiopathic scoliosis is an orthopedic deformity prevalent in the pediatric population. Despite its frequency, the exact cause remains elusive, with hypotheses largely revolving around genetic and hormonal influences. Protein biomarkers, including Gi-protein alpha subunits, fibrillin-1 and -2, and differentially expressed proteins, might contribute to muscle alterations in AIS. Concerning single-nucleotide polymorphism studies, LBX1 was the most analyzed gene, and two SNPs (rs11190870 and rs678741) exhibited a higher odds ratio for AIS relative to the control group. The genes PAX3, AJAP1, BNC2, and PAX1 were also studied, yielding mixed results for some SNPs. Persistent efforts in molecular research are crucial to the treatment of AIS before the attainment of full skeletal maturity, thereby reducing severe complications associated with postural alterations and compromised quality of life.

## Figures and Tables

**Table 1:** Main study characteristics.

Author	Participants (AIS/CTRL)	Age	Scoliosis Classification	Sample Collection	Biomarker	Results
Akoume et al. [24]	1196/395	10–16	CA > 10° CA > 40°	Osteoblast, myoblast from muscle biopsies, and peripheral blood mononuclear cells	Gαi Proteins FG1, FG2, FG3	FG1 endophenotype was represented equally in both AIS groups (*p* = 0.545); FG2 endophenotype was overrepresented in the severe AIS group (*p* = 0.0005); FG3 endophenotype was overrepresented in the moderate AIS group (*p* = 0.0001).
Buchan et al. [25]	852/669	9–18	CA > 10° CA > 40°	Paraspinal muscle biopsies	fibrillin-1 (FBN1), fibrillin-2 (FBN2)	Rare variants in FBN1 and FBN2 were enriched in severe AIS cases (7.6%) compared with CTRL (2.4%) (OR = 3.5, *p* = 0.00054); Scoliosis severity was associated with rare variants of FBN1 and FBN2 in European and (*p* = 0.0012) Chinese (*p* = 0.0376) cohorts.
Dai et al. [26]	80/50	14.3 ± 3.3	NR	Blood sample and paraspinal muscle biopsies	Dipeptidyl peptidase-4	Tissue expression of DPP-4 (*p* = 0.032) and STAT1 (*p* = 0.003) was significantly decreased in AISp than CTRL.
Gao et al. [27]	447F 66M/440	10–16	CA > 15°	Genomic DNA isolation from peripheral blood	SNPs rs11190870, rs625039, rs11598564 in LBX1	rs625039 OR = 1.49 (95% CI 1.23–1.80, *p* = 5.09 × 10^−5^); rs11190870 OR = 1.70 (95% CI 1.42–2.04, *p* = 1.17 × 10^−8^); rs11598564 OR = 1.52 (95% CI 1.27–1.83, *p* = 5.54 × 10^−6^).
Liu et al. [28]	281	15.8 ± 0.76	CA 20°–55°	Genomic DNA isolation from peripheral blood	SNPs: rs3025058 in MMP-3, rs1800795 in IL-6	MMP-3 and IL-6 were not statistically different between AISp and CTRL (*p* > 0.05).
Man et al. [29]	319/210	10–16	CA > 10°	Genomic DNA isolation from peripheral blood	SNPs: rs11190870 in LBX1, rs12946942 in SOX9/KCNJ2, rs13398147 in PAX3/EPH4, rs241215 in AJAP1, rs3904778 in BNC2, rs6570507 in GPR126, rs678741 in LBX1-AS1	rs11190870 OR = 1.56 (1.21–2.01); rs12946942 OR = 0.82 (0.66–1.18); rs13398147 OR = 0.88 (0.65–1.18); rs241215 OR = 1.11 (0.83–1.47); rs3904778 OR = 0.74 (0.57–0.96); rs6570507 OR = 1.39 (1.07–1.79); rs678741 OR = 1.67 (1.29–2.15).
Wang et al. [30]	8/50	13. 4 ± 2.1	CA < 30°	Blood sample	Muscle-related DEPs	AIM1L, SOX2, WDR7, DNM3 were lower in AISp than in CTRL; 71 more proteins were upregulated in AISp than CTRL.
Wu et al. [31]	1208/2498	13.7 ± 2.1	CA > 20°	Blood sample and paraspinal muscle biopsies	SNPs: rs687621 of ABO, rs1455114 in SOX6, rs4513093 in CDH13	rs4513093 OR = 1.20 (1.04–1.40); rs687621 OR = 0.87 (CI NR); rs1455114—no significant differences.
Xia et al. [32]	1323/1670	10–18	CA > 20°	Genomic DNA isolation from peripheral blood	SNPs: rs12517904, rs117273909 in IRX	rs12517904 OR = 1.14 (1.02–1.26) rs117273909 OR = 1.01 (0.40–2.56)
Xu et al., (2018) [33]	2914/3924 84 biopsies	12.3 ± 1.9	CA < 60° CA > 60°	Genomic DNA isolation from peripheral blood and paraspinal muscle biopsies	SNPs: rs6137473, rs169311 in PAX1	rs6137473 OR = 1.17 (1.09–1.26); rs169311 OR = 1.22 (1.14–1.30); Expression of PAX1 was significantly lower in the concave side than in the convex side (*p* < 0.001). Neither SNP was associated with curve severity.
Xu et al., (2019) [34]	1785/2680	10–16	CA > 20°	Genomic DNA isolation from peripheral blood	SNPs: rs678741 in LBX1, rs241215 in AJAP1, rs13398147 in PAX3, rs16934784 in BNC2, rs2050157 in GPR126, rs2180439 in PAX1, rs4940576 in BCL2, rs7593846 in MEIS1, rs7633294 in MAGI1, rs9810566 in TNIK	rs241215 OR = 0.75 (0.66–0.86); rs7593846 OR = 1.23 (1.09–1.39); rs13398147 OR = 1.46 (1.27–1.49); rs7633294 OR = 1.27 (1.12–1.44); rs9810566 OR = 1.30 (1.15–1.47); rs2050157 OR = 1.24 (1.10–1.41); rs16934784 OR = 1.35 (1.18–1.55); rs678741 OR = 1.42 (1.26–1.60); rs4940576 OR = 1.39 (1.23–1.57); rs2180439 OR = 1.25 (1.11–1.42).
Xu et al., (2020) [35]	40 AIS and 20 congenital scoliosis	10–18	NR	Paraspinal muscle biopsies	Genes LBX1 and PAX3, Wnt/β-catenin	AISp had lower mRNA expression of LBX1 (*p* = 0.03), PAX3 (*p* = 0.001), and Wnt/β-catenin (*p* = 0.001) in the concave side than in the convex side at the apical region.
Li et al. [36]	1725/2600 30 AIS and 20 congenital scoliosis	13.4 ± 2.2	CA > 10°	Genomic DNA isolation from peripheral blood and paraspinal muscle biopsies	SNP rs1978060 in TBX1	AISp had a significantly higher frequency of GG than CTRL. Tissue expression of TBX1 gene in AISp was lower than in congenital patients (*p* = 0.008).

AIS: Adolescent idiopathic scoliosis; CA: Cobb angle; DEPs: differentially expressed proteins; SNPs: single-nucleotide polymorphisms; CTRL: control group; NR: not reported.

## Data Availability

All data are available from the corresponding author on reasonable request.
